# Methylenetetrahydrofolate reductase (MTHFR) gene polymorphisms resulting in suboptimal oocyte maturation: a discussion of folate status, neural tube defects, schizophrenia, and vasculopathy

**DOI:** 10.1186/1743-1050-5-5

**Published:** 2008-07-10

**Authors:** Piet Hein Jongbloet, André LM Verbeek, Martin den Heijer, Nel Roeleveld

**Affiliations:** 1 Department of Epidemiology, Biostatistics, and Health Technology Assessment, Radboud University Nijmegen Medical Centre, PO Box 9101, 6500 HB, Nijmegen, The Netherlands

## Abstract

Several conditions apparent at birth, e.g., neural tube defects (NTDs) and cardiac anomalies, are associated with polymorphisms in folate-related genes, such as the 677C → T polymorphism of the methylenetetrahydrofolate reductase (MTHFR) gene. Similar associations have been established for several constitutional chronic diseases in adulthood, such as schizophrenia, cardiovascular diseases, dementia, and even neoplasias in different organ systems. This spectrum of developmental anomalies and constitutional diseases may be linked to high-risk conceptions related to preovulatory overripeness ovopathy (PrOO). Some developmental anomalies, such as NTDs, are to a large extent prevented by supplementation of folic acid before conception, but supplementation does not seem to prevent cardiovascular disease or cognitive decline. These diverging results can be elucidated by introduction of the PrOO concept, as MTHFR polymorphisms and inherent low folate levels induce both non-optimal maturation of the oocyte and unsuccessful DNA methylation and demethylation, i.e. epigenetic mutations. The PrOO concept is testable and predicts in a random population the following: (1) female carriers of specific genetic MTHFR variants exhibit more ovulatory disturbances and inherent subfecundity traits, (2) descendents from a carrier mother, when compared with those from a wild-type mother, are more frequently conceived in PrOO high-risk conditions and, thus, (3) disadvantaged in life expectancy. If so, some MTHFR polymorphisms represent a novel, genetically determined, PrOO high-risk conception category comparable to those which are environmentally and behaviorly influenced. These high-risk conditions may cause developmental anomalies and defective epigenetic reprogramming in progeny. The interaction between genetic and environmental factors is a plausible mechanism of multifactorial inheritance.

## Introduction

Most theories related to the origin of adult diseases focus on genetic causes and direct environmental effects preceding disease onset by several years at most. The view that diseases in adulthood can partly be explained by conditions earlier in life or even before birth is gaining scientific support. Previously, we proposed non-optimal oocyte ripening or impaired oocyte maturation can be an important cause of developmental anomaly and disease later in adult life [1-4]. The broad spectrum of diseases possibly related to suboptimal oocyte ripening strikingly appears to correspond with diseases that have been associated with the MTHFR 677C → T polymorphism. This genetic variant goes hand in hand with low folate and elevated homocysteine levels. We hypothesize that suboptimal maturation of the oocyte is relevant in the enigmatic relation between MTHFR variants and associated diseases.

In this paper, we review current knowledge on MTHFR polymorphisms and folate levels as they relate to developmental anomalies at birth and selected constitutional disease in adulthood. We also recapitulate the ovopathy concept and posit that the 677C → T variant and inherent low folate levels are accompanied by low estrogenisation, and that this condition induces preovulatory overripeness ovopathy (PrOO). This leads to a high-risk conception mediated by genetic factors, analogous to the environmentally and behaviorally conditioned high-risk conceptions, and an origin *ab ovo *for some congenital anomalies and constitutional diseases. Indeed, a genetic PrOO determinant emerges as an explanation for the diverging preventive effects of folate in NTDs versus adult diseases. We also discuss the relationship between low folate levels and unsuccessful DNA methylation patterns in the context of epigenetic mutations. Furthermore, testing strategies are proposed to establish the causality of the relation between MTHFR polymorphisms and PrOO conceptions in random populations.

### MTHFR-polymorphisms associated with congenital anomalies as well as with chronic diseases in adulthood and diverging benefits of folic acid supplementation

Folate is an important B vitamin that plays a pivotal role in remethylation of homocysteine to methionine, which is essential for DNA-synthesis, DNA-repair, and DNA-imprinting processes [5]. Reduction of 5,10-methylenetetrahydrofolate into 5-methyltetrahydrofolate, the predominant circulatory form of folate is catalyzed by MTHFR, the regulating key enzyme for availability of active folate at the expense of elevated homocysteine levels [6]. In 1995, the most frequently occurring polymorphism in the MTHFR gene 677C → T was identified [7]. This allele is present in heterozygous (CT) or homozygous (TT) carrier state in 40% and 5–15% of individuals [8], respectively, while the specific activity of folate and the folate metabolism is correspondingly reduced by up to 30% and 65%. In the homozygous form, this reduction is associated with a 25% increase of homocysteine levels. Thus, hyperhomocysteinemia is conditioned either genetically or nutritionally, but it can be alleviated by adequate folic acid intake.

It has become evident that the 677C → T variant as well as low-folate intake by the mother contribute to increased risks of NTDs and cardiac anomalies. The underlying pathogenic mechanism which causes this detrimental effect is not fully understood [9,10]. However, important preventive effects up to 50–75% have been effectuated for NTDs by supplementation of between 200 μg to 5 mg folic acid daily, particularly before conception [9,11] or by food fortification, as implemented in USA and Canada [12]. Over the last decade, MTHFR polymorphisms and elevated total plasma homocysteine concentrations have also been associated with a broad range of conditions in adulthood, albeit more modestly, e.g., with schizophrenia, unipolar depression, bipolar disorder [13-15], diabetic retinopathy[16], ovulatory infertility [17,18], cardiovascular disease, atherosclerosis, and thromboembolic events [19,20], renal failure [21], dementia, and cognitive impairment [22,23]. The underlying mechanisms in the pathogenesis of these chronic diseases remain still more poorly understood compared to those of developmental anomalies. However, the general opinion is that the 677C → T variant exerts its influence by ambiently elevated homocysteine levels, i.e. 'the homocysteine hypothesis' [20]. Several large-scale clinical trials evaluating vitamin supplementation were performed to reduce homocysteine concentrations with the goal to reduce cardiovascular disease and dementia, or at least to delay their onset. However, in spite of significant reductions of homocysteine for each nmol/L increase in serum folate, the results remained debatable or even negative [23-26].

An additional enigma is the marginal association between MTHFR polymorphisms and several neoplasias with diverging incidences according to age [27,28]. A meta-analysis of all published leukemia cases revealed that an association with the 677C → T allele was present in adulthood, but this effect was lost in childhood. This was the basis for suggesting a 'protective' role for this allele [29], and a similar diverging finding has been mentioned for colorectal neoplasia [30].

### The pre-ovulatory overripeness ovopathy (PrOO) concept

This concept was initially derived from animal research in the 19^th ^and 20^th ^century as well as observations in human reproduction [1-4]. Meiotic progression and optimal developmental oocyte competence in mammals occur during highly critical periods of follicle formation and ultimate oocyte maturation. The molecular, biochemical, and physiological processes in the oocyte are essential for the pleiotropic consequences in both nuclear and cytoplasmic constituents. Ideal concentrations of estrogens and optimally ripened oocytes (OptRO) are apparent during optimal conditions for reproduction. This coincides with optimal maternal age, adequate nutritional state, and post-pregnancy restoration of a regulatory ovulatory pattern, and with the seasonally-bound ovulation peaks, guaranteeing optimal embryo quality and favourable downstream effects on subsequent events, i.e., on life expectancy [1-4]. In fact, adequate estrogen concentrations in healthy women have been associated with achieving clinical pregnancy, intermediate levels with early pregnancy loss, while still lower levels were associated with non-conception cycles [31].

In contrast, abnormal estrogen concentrations cause deficient oocyte maturation which is the basis of preovulatory overripeness ovopathy (PrOO), and leads to fertilization of non-optimally matured oocytes [1-4]. The impact of oocyte attrition before fertilization is hypothesized to entail disadvantageous embryo consequences, including aneuploidy, deficient implantation, intrauterine growth retardation, prenatal loss, and developmental anomalies in various tissue systems. Inappropriate estrogen levels are physiologically conditioned and coincide with transitional stages of reproductive life in which the ovulatory pattern is most variable (i.e., at the extremes of maternal age, after very short and long pregnancy intervals, and with the seasonally-bound restoration and inhibition, i.e., breakthrough and breakdown of the ovulatory patttern). Too low and too high body mass, endocrine disruptors such as pharmaceuticals, narcotics, and toxins may also interact with estrogen levels and influence ovulatory patterns [1-3].

Excessive dysfunctional oocyte maturation results in poor quality oocytes and, after surpassing a certain threshold, in disproportionately increasing numbers of pathological conceptuses. Excess vanishing of pathological outcome will turn into excess preterm loss, and eventually in deficits of pathological births. This is called the *dose-response fallacy *in reproductive studies described by Selevan and Lemasters [32]. In our opinion, the ovopathy concept is one possible explanation for the dose-response fallacy.

### MTHFR polymorphisms and low folate levels as the genetic determinant for PrOO

**Figure 1:**
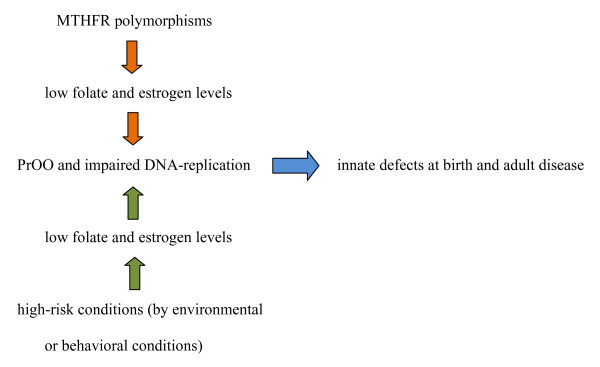
**MHTRF gene polymorphisms as well as behaviorally and/or environmentally influenced high-risk conditions cause PrOO and epigenetic DNA-alterations either independently or in combination.** This results in innate developmental defects at birth (e.g. NTDs) or in adulthood (e.g. schizophrenia).

The spectrum of developmental anomalies associated with MTHFR polymorphisms and/or folate deficiency appears analogous to the spectrum of anomalies related to PrOO induced by endocrine disturbances during the transitional stages of reproductive life and/or by divergent reproductive behavior, e.g. unusual maternal age or pregnancy interval [3] (see Figure 1). Low folate serum levels in Rhesus monkeys have been associated with granulosa cell impairment and with decreased estradiol and progesteron levels. Reduction of follicle growth and delayed ovulation are markers for retardation of embryonic growth and malformation [33]. Folate levels are also essential for sperm maturation, as inadequate folate intake is inversely associated with overall frequencies of several types of aneuploid sperm in healthy men [34]. The MTHFR CT and TT genotypes with inherent low folate status are candidates for compromising oocyte maturation, and may set the stage for a genetically conditioned high-risk conception analogous to those resulting from endocrine disturbances induced by environmental or behavioral conditions (see Figure 1.).

This genetically conditioned high-risk for PrOO explains several unexplained phenomena related to MTHFR variants as well as to low dietary folate. Both conditions are associated with increased fetal loss, intrauterine growth retardation, and heart defects in female mice [35], as well as with women experiencing fetal aneuploidy, recurrent pregnancy loss, early and late pregnancy loss, preeclampsia, preterm premature rupture of membranes, and of particular interest, congenital anomalies [36-39]. These reproductive casualties have been related to non-optimally matured oocytes and aberrant blastocyst nidation [1-4]. The probability for embryos with TT or CT genotypes to arrest at an early stage has been advanced as an explanation for the 'unique distribution' of the 677CT and 677TT genotypes in spontaneously aborted embryos, irrespective of chromosomal integrity [40-43]. This is in line with the *dose-response fallacy *being inherent to the PrOO concept. Other findings are also in accordance with disproportionate levels of oocyte deterioration and a *dose-response fallacy*: a dose-specific reduced risk of progeny with cleft lip with or without cleft palate(CL/P) from mothers carrying either one or two copies of the 677C → T variant (RRs: 0.71 and 0.38, respectively), a negative association for children with CL/P (RRs: 1.05 and 0.74) versus a positive one for cleft palate only (CPO; RRs: 2.06 and 1.75), and a fourfold increased risk of orofacial clefts in mothers using folic acid [44].

The finding of MTHFR 677C → T mutations in the mother (but not in children with congenital heart defects) [45] is in general agreement with PrOO as a primary cause of developmental anomalies [46]. Additionally, teratogenic effect appears to be dose-specific in NTDs with 60% heterozygous and 90% homozygous mothers [9], and is also apparent from the increasing male sex preponderance among randomly selected newborns according to maternal MTHFR wild-type, heterozygous, and homozygous carriership: 47%, 50%, and 67% boys, respectively [47]. This sex ratio modulation offers further support for the PrOO concept [4].

It should be noted that periconceptional multivitamin use including folic acid reduces the dose-response risk of preeclampsia and ovulatory infertility [17,18]. In IVF treatment, it results in better embryo quality (defined by cell number), embryo fragmentation rate, or short-term pregnancy outcomes [48]. In contrast, the disappointing curative effect of folate supplements for constitutional diseases associated with MTHFR polymorphisms [23-26] is in line with developmental weaknesses *ab ovo*. In other words, preventive measures are effective only before conception but not afterward, and that threatened oocytes may be the culprit of specific constitutional diseases encountered long after birth.

The conjecture of a common origin *ab ovo *for both developmental anomalies and constitutional disease is consistent with the environmental determinants related to non-optimally maturing oocytes [1-4]. This is in particular evident by the sesonally-bound month-of-birth configurations observed in NTDs and cardiac anomalies [1,3,48,49] but equally evident for schizophrenia, eating disorders, subfecundity, DM-1 and DM-2, and childhood leukemia [50-54]. Analogous month-of-birth configurations were also present in three consecutive samples of childhood leukemia in the Netherlands [personal communication]. Interaction between MTHFR genetic determinants, nutrition, and other maternal reproductive determinants should be emphasized in these disease processes, as illustrated in Figure 1.

### MTHFR genes, low folate and epigenetic reprogramming

As mentioned previously, adequate folate levels are a prerequisite not only for optimal oocyte maturation but also for remethylation of homocysteine to methionine – the key epigenetic contributor to gene activation and/or reprogramming [6]. These epigenetic processes are essential to confer stability of gene expression during mammalian development and necessary for correct initiation of embryonic gene expression and early lineage development in the embryo. Such epigenetic modulation profoundly influences transcriptional repression, chromatin structure, X-inactivation, and allelic imprinting and silencing.

Incorrect DNA reprogamming results in epigenetic mutation, which does not follow the classic rules of Mendelian inheritance. Active transport of methionine happens in all animal and human oocytes and in early preimplantation embryos. DNA-methylation is concentrated at specific stages when developmental potency of cells changes, i.e., during the final phase of oocyte maturation. In contrast, DNA-demethylation occurs immediately after fertilization [5,55,56]. It follows that maternal reproductive factors can affect fetal development via epigenetic modifications of DNA [57]. Interestingly, ovarian hyperstimulation was the common factor in all twelve reproductive histories of women who gave birth to offspring affected with Beckwith-Wiedemann syndrome [58], and assisted reproduction technology [55,56] and PrOO [59] have been associated with other imprinting disorders.

The combined effect of PrOO and epigenetic mutations evoked by MTHFR variants (and low folate levels) can explain many developmental puzzles in animals and humans. For example, epigenetic modifications in lambs derived from oocytes retrieved from ewes given a vitamin B_12 _and folate restricted diet during the periconceptional period, resulted in obesity, insulin resistance, hypertension, and altered immune responses when full grown [60]. This was especially observed in rams, itself a noteworthy finding regarding sex ratio modulation by PrOO [4]. Additionally, pre-conception dietary methyl supplements have been shown to alter the capacity for methylation and expression of the imprinted Agouti gene and strongly affect the phenotype and long-term health of the young in female Agouti mice [61]. Furthermore, insults to the oocyte appear to be responsible for aberrant epigenetic reprogramming events at the zygote stage and even demethylation of the paternal pronucleus in mice seems maternally driven [62]. A number of pathologic conditions have been associated with decreased global methylation, including spina bifida [63], schizophrenia [64], and certain neoplasias [30,65,66].

### MTHFR polymorphisms related to the PrOO concept: testing the hypothesis

**Figure 2:**
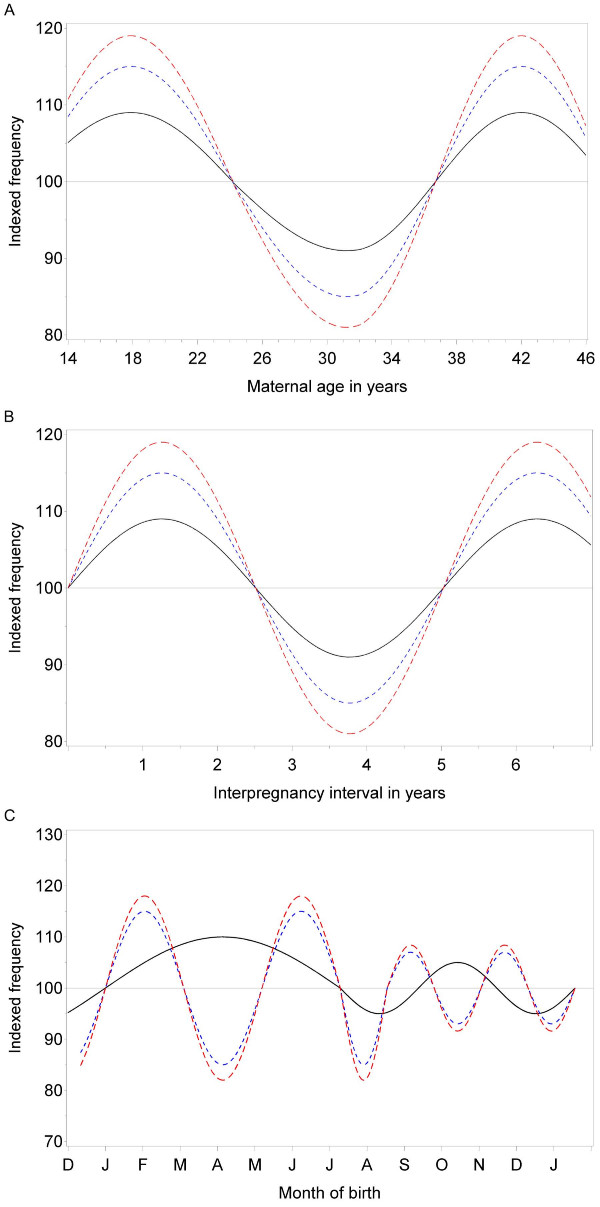
**The indexed birth incidence of descendents (average=100) from wild-type MTHFR gene mothers is expected to be conform that in a random population [solid line, black] during (2a) reproductive life), (2b) interbirth interval and (2c) winter and summer birth peak.** The PrOO concept predicts disproportional increases of births from heterozygous [interupted line, blue] – and more excessively – from homozygous [interrupted line, red] MTHFR allele carrier mothers. This will occur at (2a) menarche and menopause, (2b) after parturition or long fallow period and (2c) at the onset and the end of the winter and summer birth peak. A dose-response fallacy may be expected at the extremes of maternal age and interbirth interval (2a and 2b) or at the birth troughs (2c).

MTHFR polymorphism status in females is hypothesized to entail a genetically determined propensity to PrOO, which may be likened to the high-risk conceptions elicited by environmental and behavioral conditions. The variant alleles operate either independently or in concert and may strengthen each other. Therefore, we predict that:

1) Heterozygous and homozygous 677C → T carrier females suffer from subfecundity traits in a dose-response manner when compared to females with no mutation (CC). Apart from giving birth to affected progeny, they also experience more menstrual disorders, longer time to achieve pregnancy, longer interpregnancy intervals, and more pronounced reproductive seasonal variation.

2) Offspring from homozygous (and to a lesser extent from heterozygous 677C → T carrier mothers), being genetically more susceptible to environmental triggers than mothers without this mutation, are more frequently conceived in high-risk conditions characterized by ovulatory disturbances. Typical PrOO characteristics are depicted in Figure 2, and include a U-shaped birth distribution related to (2a) maternal age and (2b) interbirth interval, and (2c) a disproportionate seasonally-bound month-of-birth distribution [1-3].

3) Descendents from homozygous (and to a lesser extent from heterozygous 677 → T carrier women) have reduced life expectancy compared to offspring from mothers without this polymorphism.

These effects may be obscured by early loss before birth as a consequence of preterm premature rupture of membranes and eclampsia associated with MTHFR polymorphisms [36,38] or due to untimely death, often before diagnosis, or due to other poor outcomes related to MTHFR polymorphisms. As this may cause spurious negative associations, as e.g., in childhood leukemia [29], these effects are age-specific and more apparent in younger than in older individuals, as has been demonstrated [personal communication]. Disproportionate levels of oocyte deterioration and *dose-response fallacy *will in particular occur at the extremes of maternal age, birth interval or at the seasonal transitions (Figure 2a, 2b and 2c).

In **conclusion**, MTHFR polymorphisms and resulting low folate levels warrant consideration as factors inducing non-optimally matured oocytes before conception. They represent a novel, genetically determined, high-risk PrOO condition comparable to the endocrine disturbances elicited by environmental and behavioral conditions. Further study of the interaction between genetic and environmental factors may indentify mechanisms of multifactorial inheritance and explain many commonly associated enigmata in chronic constitutional diseases.

## Competing interests

The authors declare that they have no competing interests.

## Authors' contributions

The content of this manuscript was subject of the fare-well lecture of PHJ on 12^th ^of June 2007 at the Department of Epidemiology, Biostatistics, and Health Technology Assessment at the Radboud University Nijmegen, the Netherlands. The co-authors AV, MdH, and NR participated in the design of the study and the coordination for the manuscript preparation. They revised it critically for important intellectual content and interpretation of the arguments. All authors read and approved the final manuscript.
